# RhoGDI2 up-regulates P-glycoprotein expression via Rac1 in gastric cancer cells

**DOI:** 10.1186/s12935-015-0190-4

**Published:** 2015-04-15

**Authors:** Zhong Zheng, Bingya Liu, Xiaohua Wu

**Affiliations:** Department of Gynecologic Oncology, Fudan University Shanghai Cancer Center, Department of Oncology, Shanghai Medical College, Fudan University, 270 Dongan Road, Shanghai, 200032 People’s Republic of China; Department of Surgery, Shanghai Key Laboratory of Gastric Neoplasms, Shanghai Institute of Digestive Surgery, and Gastroenterology, Ruijin Hospital, Jiaotong University School of Medicine, Shanghai, China

**Keywords:** RhoGDI2, Multidrug resistance, Gastric cancer, P-gp, Rac1

## Abstract

Multidrug resistance (MDR) is a major clinical obstacle in treatment of gastric cancer. Previously, using 2D electrophoresis-mass spectrometry, we identified RhoGDI2 as a contributor to 5-FU resistance in colon cancer cells, and also confer gastric cancer cells resistance to 5-FU. Here, we reported RhoGDI2 also induced MDR in gastric cancer cell line (MKN-45). To explore the underlining mechanism, we detected the mRNA, protein expression, activity of P-glycoprotein (P-gp) in MKN-45 stably transfected with RhoGDI2 expressing or control vector. All the mRNA, protein level, activity were increased by 130%, 230%, 35% respectively after ectopic expression of RhoGDI2. RhoGDI2 was correlated with P-gp expression in gastric cancer tissues as detected by immunohistochemistry. To further study how RhoGDI2 up-regulates P-gp expression, we tested the activity of Rac1 in MKN-45/RhoGDI2 and MKN-45/GFP. Ectopic expression of RhoGDI2 increased Rac1 activity (P < 0.05). For more important, silencing of Rac1 expression by siRNA decreased P-gp expression to undetectable level. Overall, these findings suggest that RhoGDI2 up-regulates P-gp expression via Rac1 to induce MDR.

**Electronic supplementary material:**

The online version of this article (doi:10.1186/s12935-015-0190-4) contains supplementary material, which is available to authorized users.

## Introduction

Gastric cancer is the fourth most common type of cancer and the second leading cause of cancer-related deaths in the world [1]. Chemotherapy plays an important role in the treatment of gastric cancer both in adjuvant and advanced settings. However, the efficacy of chemotherapy for gastric cancer is limited due to insensitivity and the development of multi-drug resistance (MDR). Numerous efforts have been made to understand the mechanisms underlying MDR. [2] It is known that MDR involves a large number of molecules and complex mechanisms. Classical drug-resistant molecules, such as P-glycoprotein (P-gp)/ABCB1 and multi-drug resistance protein (MRP1)/ABCC1, have been found to play important roles in mediating MDR in some gastric cancers [3]. To reveal the mechanisms underlying drug resistance, we previously compared the proteomic profiles of 5-FU resistant and sensitive colon cancer cells by 2-D gel electrophoresis [4]. We found that RhoGDI2 was up-regulated in 5-fluorouracil (5-FU) resistant colon cancer cells (LoVo/5-FU) and that the knockdown of RhoGDI2 expression by transfection with RhoGDI2-specific siRNA significantly increased sensitivity to 5-FU in LoVo/5-FU [4]. These data suggested that RhoGDI2 confers resistance to 5-FU in colon cancer cells. Later on, our and other groups also showed RhoGDI2 play important role in multi-drug resistance in gastric cancer [5-9].

RhoGDI2 belongs to a family of Rho GTPase dissociate inhibitors (GDIs). GDIs are pivotal regulators of Rho GTPase function typified by forming a complex with Rho GTPase, modulating their nucleotide exchange and membrane association. Therefore, they play an important role in regulating the actin cytoskeleton, cell polarity, microtubule dynamics, membrane transport pathways, and transcription factor activity [10,11]. Unlike other members (RhoGDI1, RhoGDI3), RhoGDI2 is preferentially expressed in hematopoietic cells and overexpressed in gastric cancer it also appears to have a narrow selectivity and lower binding affinity for Rho GTPases [12]. RhoGDI2 associates with and negatively regulates Rac1 and Rac3 in breast cancer cells, but not RhoA, Cdc42, and RhoC [13], whereas it positively regulates Rac1 in human bladder cancer cells [14].

RhoGDI2 is also a substrate for caspases and becomes cleaved in various cell types during apoptosis. Recently, several lines of study reported the important role of RhoGDI2 in multi-drug resistance in gastric cancer [4-9]. A Korea group showed RhoGDI2 confer gastric cancer cells resistance to cisplatin induced apoptosis by several mechanisms. VEGF-C [7], 14-3-3σ [8], PLCγ [5], Bcl-2 [6] may mediate the effects of RhoGDI2 in drug resistance. However, our previous study showed a different mechanism [9]. RhoGDI2 reverted the low dose 5-FU-induced G2/M arrest rather than high dose 5-FU induced apoptosis [9]. Here we reported another new mechanism by which RhoGDI2 induced MDR, which is that RhoGDI2 upregulates P-gp expression via Rac1.

## Results

### Ectopic expression of RhoGDI2 up-regulates P-gp expression

**Figure 1:**
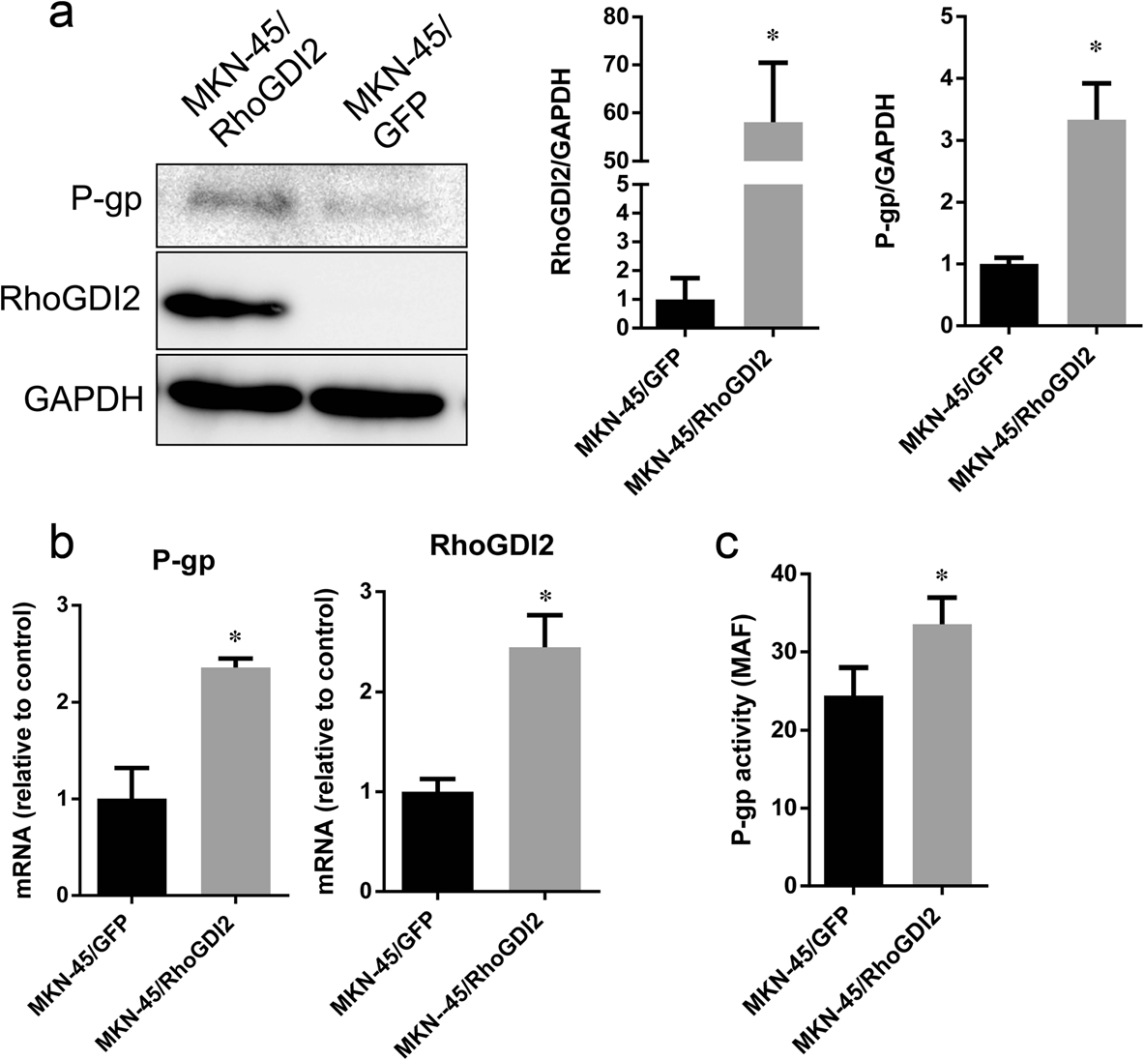
Ectopic expression of RhoGDI2 increased P-gp mRNA, protein and activity. **(a)** Western blotting analysis of RhoGDI2 and P-gp expression in MKN-45/RhoGDI2 and MKN-45/GFP. The immunoblot image was quantitated by densitometric analysis (right) (n = 3 independent experiments). *p < 0.05 vs MKN-45/GFP. **(b)** The mRNA of RhoGDI2 (left) and P-gp (right) in MKN-45/RhoGDI2 and MKN-45/GFP was detected by RT-PCR. **(c)** P-gp activity in MKN-45/RhoGDI2 and MKN-45/GFP was measured as described in materials and methods, MAF was plotted. Data are expressed as mean ± SD from three independent experiments; *p < 0.001 vs MKN-45/GFP.

To determine the role of RhoGDI2 in drug resistance, we established a gastric cancer cell line (MKN-45/RhoGDI2) stably transfected with a RhoGDI2 expression vector. Ectopic expression of RhoGDI2 in MKN-45 increased RhoGDI2 protein and mRNA expression by 58 and 1.6 fold respectively (Figure 1a and b, both P < 0.01). The previous study showed ectopic expression of RhoGDI2 confer MKN-45 resistance to 5-FU. In addition to that, the IC50 of doxorubicin, Paclitaxel was increased in MKN-45 from 0.23 ± 0.011μM to 0.31 ± 0.025μM (*P* < 0.05) and from 4.9 ± 0.33 to 8.5 ± 0.60nM (*P* < 0.05) respectively after ectopic expression of RhoGDI2. To investigate the mechanisms underlying RhoGDI2 induced multi-drug resistance, we compared the mRNA levels of several MDR related genes expression by RT-PCR including Mdr-1, multi-drug resistance protein 1 (MRP-1), MRP-2, MRP-3, MRP-4, MRP-5, MRP-8 between MKN-45/RhoGDI2 and MKN-45/GFP (Additional file 1 Figure S1). The levels of Mdr-1 (Figure 1b) mRNA was the only one significantly increased in MKN-45/RhoGDI2 by 1.3 fold. Protein level of *Mdr-1* gene (P-gp) was increased by 2.3 fold in MKN-45/RhoGDI2, comparing with MKN-45/GFP (Figure 1a, *P* < 0.05). In addition to that, the P-gp activity of MKN-45/RhoGDI2 was also increased by 32.5%, comparing with MKN-45/GFP (Figure 1c, *P* < 0.05).

### RhoGDI2 correlates with P-gp expression in human gastric cancer tissue

**Figure 2:**
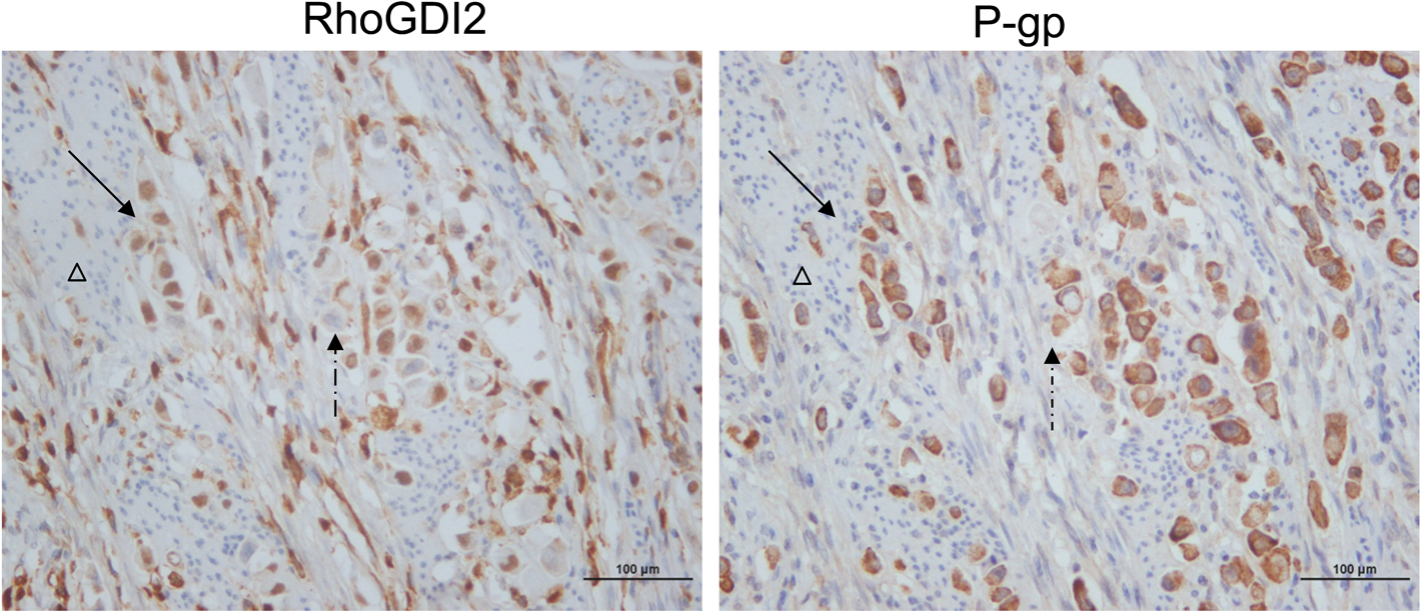
Representative images of IHC staining with anti-P-gp and anti-RhoGDI2 demonstrate the co-expression of P-pg and RhoGDI2 in serial sections of gastric cancer tissue. Serial sectioning slices were stained with anti-P-gp and anti-RhoGDI2 from one case. Brown color represent positive of staining, the negative staining was contra-stained with hematoxylin and shown in blue color. Most of the cancer cells were positively stained with both anti-P-gp and anti-RhoGDI2. RhoGDI2 is expressed majorly in nuclear, whereas P-gp majorly presents in membrane. Some of the cancerous cells expressed high RhoGDI2 also were positively stained with anti-P-gp (shown in solid arrow), whereas some of the cancerous cells with lower RhoGDI2 were also weakly stained with anti-P-gp (shown in dashed arrow). The non-cancerous cells are negative for both P-gp and RhoGDI2 (marked with triangle). Magnification 400×. Scale bar = 100 μm.

To determine whether there was a correlation between the expression of RhoGDI2 and P-gp in patients with gastric cancer, RhoGDI2 and P-gp levels in gastric cancer tissues were analysed by IHC. As shown in previous study, RhoGDI2 was overexpressed in gastric cancer cells [9]. P-gp was present in the membrane of both cancerous and adjacent benign gastric epithelial cells. There was no significant difference of the P-gp levels between cancer and benign tissues. Interestingly, the expression of RhoGDI2 was correlated with P-gp expression only in cancerous tissue (*p =* 0.011, χ^2^ = 7.46). Co-expression of RhoGDI2 and P-gp was also frequently observed in cancer cells (Figure 2).

### RhoGDI2 up-regulates P-gp expression via Rac1

**Figure 3:**
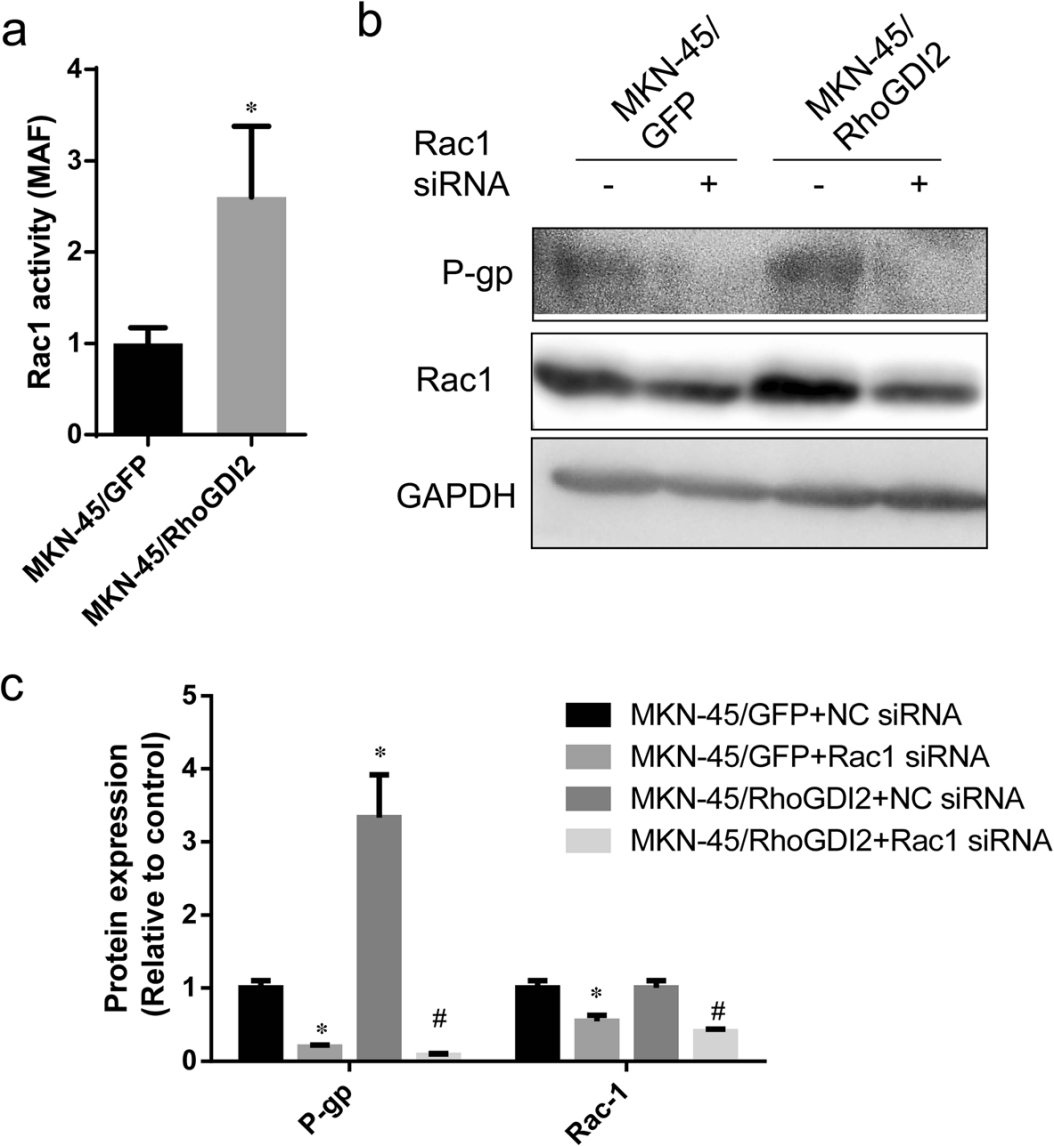
RhoGDI2 up-regulates P-gp expression via Rac1. **(a)** Rac1 activity in MKN-45/RhoGDI2 and MKN-45/GFP was measured as described in materials and methods. Data are expressed as mean ± SD from three independent experiments; *p < 0.001 vs MKN-45/GFP. **(b)** Western blot analysis of P-gp and Rac1 expression in MKN-45/RhoGDI2, MKN-45/GFP transfected with Rac-1 or Mock siRNA (pictures are representative of three independent experiments). The immunoblot image was quantitated by densitometric analysis **(c)** (n = 3 independent experiments). Data was shown in folds relative to MKN-45/GFP + NC siRNA. *p < 0.05 vs MKN-45/GFP + NC siRNA, #*p* < 0.05 vs MKN-45/RhoGDI2 + NC siRNA.

It is reported the RhoGDI2 is associated with Rac1 and regulates its activity [13-17]. Therefore, we tested the Rac1 activity in MKN-45/RhoGDI2 and MKN-45/GFP by ELISA based quantification of active Rac1 (Rac1-GTP) level. The Rac1 activity was increased in MKN-45/RhoGDI2 by 1.6 fold, comparing with MKN-45/GFP (Figure 3a, *P* < 0.05). Next, we test whether Rac1 is required for up-regulation P-gp by RhoGDI2 overexpression. Transfection of Rac1 siRNA for 48 hours decreased Rac1 expression in both MKN-45/RhoGDI2 and MKN-45/GFP cells (Figure 3b). P-gp level was also significantly decreased after silencing of Rac1 expression in both MKN-45/RhoGDI2 and MKN-45/GFP cells (Figure 3b). It indicates the effects of RhoGDI2 induced P-gp upregulation was abolished by inhibition of Rac1.

## Discussion

In our previous study using 2D electrophoresis-mass spectrometry, we found that RhoGDI2 was a contributor to 5-FU resistance in colon cancer [4]. Later on, we showed that RhoGDI2 also confers resistance to 5-FU in gastric cancer cells [9]. Here we reported a new mechanism by which RhoGDI2 induces multidrug resistance. It is that RhoGDI2 up-regulates P-gp expression via Rac1.

Accumulative evidence show that RhoGDI2 confer multi-drug resistance in ovarian cancer, gastroenterologic cancer [4-9], breast cancer [13]. It was shown that RhoGDI2 was over-expressed in chemo-resistant fibrosarcoma cells and paclitaxel-resistant ovarian cancers, respectively [18,19]. Hee et al. [6] reported that RhoGDI2 confers resistance against multiple chemotherapeutic agents (cisplatin, etoposide, and staurosporin) -induced apoptosis in gastric cancer cells. Together with our results, we conclude that high levels of RhoGDI2 expression are associated with chemotherapy resistance in certain types of cancers, like gastroenterologic cancer.

P-gp, the product of the *ABCB1* (*mdr-1*) gene, is a full transporter comprised of 12 transmembrane segments divided into TM domains, each linked with an ATP-binding domain [20]. It was first identified due to its overexpression in drug-resistant tumor cells, where it functions as a broad range drug transporter, thereby conferring resistance to many important chemotherapeutic agents including vinblastine, doxorubicin, and paclitaxel [20,21]. Here, we reported that ectopic expression of RhoGDI2 increased P-gp expression and activity in gastric cancer. Furthermore, in patients with gastric cancer, the expression of RhoGDI2 was also positively associated with P-gp expression in cancer cells. Our previous study showed RhoGDI2 reverted the low dose 5-FU-induced G2/M arrest. Taken all these together, RhoGDI2 confers multi-drug resistance in gastric cancer by multiple mechanisms. Rho GTPase plays important roles in regulation of astral microtubules and the interaction of spindle microtubules with chromosomes during mitosis. It is reported inhibition of Rho GTPase causes a mitotic arrest. Here, we show RhoGDI2 up-regulates P-gp transcription via Rho GTPase (Rac1). Hence it is possible the versatile Rho GTPase contributes to the multiple mechanisms underlining RhoGDI2 induced MDR.

Rac1 was recognized as the important co-operator of RhoGDI2 and mediator of its function [16]. Unlike other member of Rho GTPase, RhoGDI2 preferentially binds to Rac1 and affects its activity [16]. A recent study showed RhoC was also regulated by RhoGDI2 [22]. However, numerous studies have shown the conflicting role of RhoGDI2 in the regulation of Rac1 dependenting on the tumour types and/or cellular microenvironment. it is demonstrated that RhoGDI2 inhibited Rac1 activity in MDA-MB-231 human breast cancer cells [13] and mouse embryonic fibroblasts [23], whereas others showed RhoGDI2 acted as a positive regulator of Rac1 in T24 and UMUC3 human bladder cancer cells [14], H9c2 cardiomyoblast cells [17] and ovarian cancer. Both Cho et al. and our results demonstrated that RhoGDI2 up-regulated Rac1 activity [7] in gastric cancer cell lines. The mechanisms by which RhoGDI2 regulates Rac1 aren’t fully understood. Boulter et al described an evolutionarily conserved mechanism by which RhoGDI1 controls the homeostasis of Rho proteins in eukaryotic cells [24]. They showed that depletion of RhoGDI1 led to destabilization and degradation of unbound RhoGTPases resulting in a reduction of GTPase protein levels, while unexpectedly activating the remaining membrane bound fraction [24]. It isn’t clear whether RhoGDI2 function in a similar manner as RhoGDI1 to protect Rho family GTPases from degradation. Huang et al [17] reported in cardiomyoblast cell line H9c2 unlike RhoGDI1, RhoGDI2 up-regulates Rac1 mRNA transcripts, rather than Rac1 protein stability. Our and other studies didn’t show alteration of RhoGDI2 expression could results in Rac1 protein level change in cancer cells [7,13,14]. Therefore it is expected RhoGDI2 might act in a different way to regulate Rac1. Extraction Rho GTPases from the membrane by RHOGDIs is the second mechanism to regulated RHO GTPase activity [16,25]. In Breast cancer cells MDA-MB-231, knockdown of RhoGDI2 results in Rac1 translocation from the cytosol to cellular membrane compartments, leading to constitutive Rac1 activation and cell growth inhibition [13]. In contrast in bladder cancer, RhoGDI2 activated Rac activity without affecting Rac membrane/cytosol ratios [14]. However, point mutants of RhoGDI2 that increase or decrease the affinity of RhoGDI2 for GTPases abolished its ability to activate Rac [26]. It indicates there is a mechanism distinct from inhibition of membrane association involved in activation of Rac1 by RhoGDI2. Recently, using system biological method, a model was constructed to reveal how RhoGDIs act as positive regulators of Rho GTPase [27]. It indicates RhoGDIs positively regulate Rho GTPase signaling primarily by interacting with GAPs and may participate in the switching between transient and sustained signals of the Rho GTPases. It need further study to confirm whether and which GAPs are involved in our study.

In conclusion, we suggest that there is a new mechanism by which RhoGDI2 contribute to multi-drug resistance in gastric cancer cells. It may effects drug efflux by up-regulation of P-gp expression via Rac1, rather than disturbance the apoptotic pathway as shown by other group.

## Material and methods

### Cell culture

Human gastric cancer cell lines MKN-45 were obtained from the Chinese Academy of Sciences Cell Bank of Type Culture Collection (Shanghai, China). All the gastric cancer cell lines were cultured in RPMI-1640 (Invitrogen, Carlsbad, CA) supplemented with 10% FBS (PAA Laboratories GmbH, Morningside, QLD, Australia).

### Patients and tissues

Human gastric cancer tissue arrays were obtained from Outdo Biotech (Shanghai, China) with 88 individual cases of gastric cancer and matched normal colon tissue. The ethics committee at Ruijin Hospital approved the use of these tissues for research purposes.

### RhoGDI2 expressing plasmid

To generate a RhoGDI2 expression plasmid, the full-length coding region of RhoGDI2 cDNA was amplified using the primers RhoGDI2-forward CATA*CTCGAG*CGG ACA GAG ACG TGAA GCAC and RhoGDI2-reverse CACT*GGATCC*GAGT GAC AGG GTG GGA AAAG (the restriction sites of XhoI and BamHI were italic) and inserted into pIRES2-EGFP (Clontech Laboratories, Mountain View, CA) at XhoI and BamHI sites. MKN-45 cells were transfected with pIRES2-EGFP-RhoGDI2 or pIRES2-EGFP using Lipofectamine 2000 reagent (Invitrogen, Carlsbad, CA, USA) according to the manufacturer’s instructions. Stable plasmid-transfected clones were selected using 800 μg/ml G418 (Invitrogen) for 2 weeks, isolated colonies were picked up with tips, and the cells were further cultured in the presence of 400 μg/ml G418. MKN-45 cells transfected with pIRES2-EGFP-RhoGDI2 were named MKN-45/RhoGDI2 cells. MKN-45 cells transfected with pIRES2-EGFP were named MKN-45/GFP cells.

### In vitro cytotoxicity assay

The MTT assay was used to determine the relative sensitivity of cell lines to 5-FU (Xudonghaipu Pharmaceutical Co., Shanghai, China), as described previously [4]. For cell lines, cells plated in 96-well microplates were cultured with growth medium or treated with serial dilutions of 5-FU for 72 hours. Viable cells were measured with MTT (Sigma, St. Louis, MO) and the results were expressed relative to the absorbance of cells grown in the absence of drug. IC50 values were calculated by nonlinear regression analysis from triplicate independent experiments.

### Western blot analysis

Western blot analysis was done as previously described [28]. Briefly, cell lysates were separated by SDS-PAGE and transferred to a PVDF membrane. The blot was then probed with anti-RhoGDI2 (LabVision, Fremont, CA) with a dilution of 1:1000 , anti-P-gp (Santa Cruz Biotechnology, Santa Cruz, CA) with a dilution of 1:250, anti-Rac-1 (Sangon Biotech, Shanghai, China ) with a dilution of 1:500, or anti-ABCC-1 (MRP1) (Sangon Biotech, Shanghai, China ) with a dilution of 1:250 followed by an incubation with a horse radish peroxidase-conjugated secondary antibody. The signal was detected using enhanced chemiluminescence (Millipore). The expression level was quantified using Image J program (NIH).

### Immunohistochemical staining

Immunohistochemical staining was done as described previously using a DAKO EnVision + System HRP [29]. RhoGDI2 polyclonal antibody from LabVision (Fremont, CA) was applied at a 1:2,000 dilution overnight at 4°C, while anti-P-gp was applied at a 1:200 dilution. Purified rabbit-IgG was used as an isotype control. The stained sections were reviewed by two independent observers who had no prior knowledge of the clinic pathologic data of the patients. A scoring method was used as reported previously based on the fact that the specimens clearly showed varying degrees of staining intensity and percentage of cell staining [30]. Briefly, strong-intensity staining was scored as 3, moderate as 2, weak as 1, and negative as 0. For each intensity score, the percentage of cells with that score was estimated visually. A combined weighted score consisting of the sum of the percentage of cells staining at each intensity level was calculated for each sample. The immunolabelling was categorised as negative (score > 30) or positive (score ≤30) for all the tissues.

### P-gp activity (eFluxx-ID Gold uptake assay)

Fluorescent probe eFluxx-ID Gold was used to monitor P-gp functionality. Cells were trypsinized and incubated for 30 min at 37°C in phenol red free Opti-MEM medium (Invitrogen) with eFluxx-ID Gold (ENZO Life Sciences, Lörrach, Germany) according to the manufacturer’s protocol. Cells were analyzed by flow cytometry (FACSCanto; BD Biosciences, Heidelberg, Germany). Each flow cytometry analysis consisted of a record of 100,000 cells. The uptake of eFluxx-ID Gold of EGFP-positive cells was measured in the FL2 (PE) and FL1 (FITC) channel. Dead cells were excluded by using scatter parameters. In parallel experiments, P-gp inhibitor Verapamil was added to the cells to a final concentration of 20 μM for 30min during the incubation with eFluxx-ID Gold in order to determine whether the alterations in eFluxx-ID Gold uptake induced by transfection with P-gp. Multidrug resistance activity factor (MAF) was calculated as MAF (MDR1) = 100 × (fluorescence intensity with P-gp inhibitor - fluorescence intensity without P-gp inhibitor)/ fluorescence intensity with P-gp inhibitor.

### Rac1 activity assay

For measurement of Rac1 activation in cell lysates from MKN-45 cells, equal amounts of protein per sample (determined by use of a Protein Assay Kit from Bio-Rad Laboratories; Hercules, CA) were analyzed using the specific Rac1 G-LISA™ Activation Assay Kit (Cytoskeleton; Denver, CO) according to the manufacturer’s instructions. This luminescence G-LISA™ is an ELISA based assay that allows measuring the GTP-bound (active) form of small G-proteins. Active Rac1 levels were expressed as fold-increase over the active Rac1 levels in control conditions.

### Reverse Transcription-Polymerase Chain Reaction (RT-PCR) for the Detection of P-gp RhoGDI2 Expression at the mRNA Level

Expression of human P-gp and RhoGDI2 at the mRNA level in the cell lines used was verified by reverse transcription of RNA followed by polymerase chain reaction. RNA was isolated using the Trizol reagent (Invitrogen Life Technologies, Carlsbad, CA, USA). First-strand cDNA synthesis was performed with the First-Strand cDNA synthesis kit for RT-PCR (Jrdun biotechnology, Shanghai, China) with oligo(d T) primers according to the manufacturer’s instructions. Primers used for the amplification of human P-gp were 5′- CGCTGTTCGTTTCCTTTAG -3′ (sense) and 5′- CTTCTTTGCTCCTCCATTG -3′ (antisense). For RhoGDI2, primers were 5'-TTCTTCACCGACGATGAC-3' (sense) and 5'-GGAAATGTG GCAGTGTTG-3' (antisense). For MRPs, see Additional file 2 Table S1. All primers were synthesized by MWG Biotech AG (Shanghai, China). PCR was performed on the ABI Prism 7300 in a total volume of 50 μl using the Jrdun biotechnology FQ-PCR kit (Shanghai, China).

### Statistical analysis

Statistical analyses of data were performed by using the Student t-test or one-way analysis of variance, depending on the number of groups in comparison. Data that failed the test for normal distribution or homogeneous variance were analysed using the Mann–Whitney U or the Kruskal–Wallis tests. The correlation between RhoGDI2 and P-gp expression was analysed using the Fisher exact test. The statistical software SPSS version 14.0 was used for analysis. Significance was set at P < 0.05.

## Additional files

Additional file 1: Figure S1. A. The mRNA of RhoGDI2 (left) and P-gp (right) in MKN-45/RhoGDI2 and MKN-45/GFP was detected by RT-PCR. Data was expressed as relative to GAPDH (mean ±SD) from three independent experiments; **p* < 0.05 vs MNK-45/GFP. B. Western blotting analysis of MRP-1 expression in MKN-45/RhoGDI2 and MKN-45/GFP. Additional file 2: Table S1. The primers for MRPs.

## Abbreviations

MDR: Multidrug resistance
GDIs: Rho GTPase dissociate inhibitors
P-gp: P-glycoprotein
MRP: Multi-drug resistance protein
5-FU: 5-fluorouracil
