# Supplementary figures and images for: RhoGDI2 up-regulates P-glycoprotein expression via Rac1 in gastric cancer cells
Source: Cancer Cell Int. 2015 Apr 15;15:41. doi: 10.1186/s12935-015-0190-4 (PMC4404694; doi:10.1186/s12935-015-0190-4)

A

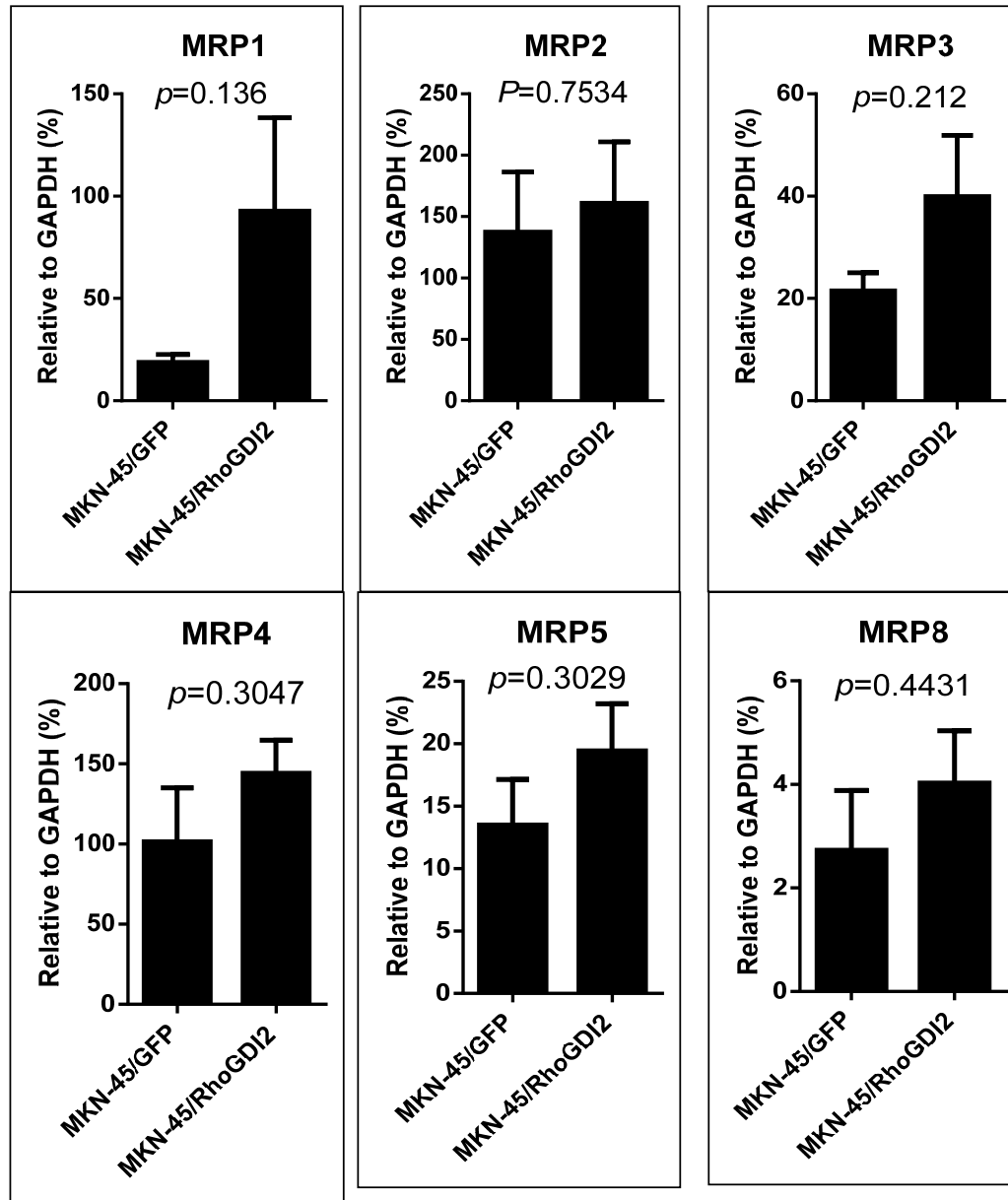

B

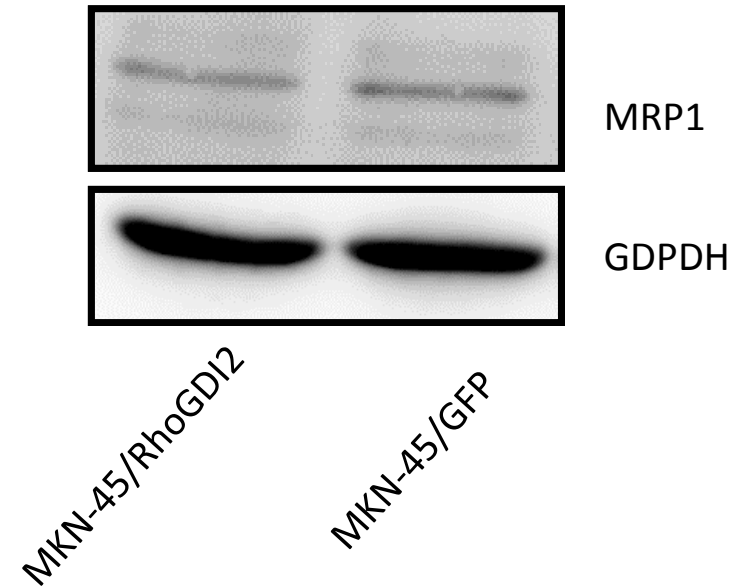

Supplemental Figure 1

Supplement: Additional file 1: Figure S1. — A. The mRNA of RhoGDI2 (left) and P-gp (right) in MKN-45/RhoGDI2 and MKN-45/GFP was detected by RT-PCR. Data was expressed as relative to GAPDH (mean ±SD) from three independent experiments; *p < 0.05 vs MNK-45/GFP. B. Western blotting analysis of MRP-1 expression in MKN-45/RhoGDI2 and MKN-45/GFP. [file 12935_2015_190_MOESM1_ESM.pdf]
