# Supplementary material for: RhoGDI2 up-regulates P-glycoprotein expression via Rac1 in gastric cancer cells
Source: Cancer Cell Int. 2015 Apr 15;15:41. doi: 10.1186/s12935-015-0190-4 (PMC4404694; doi:10.1186/s12935-015-0190-4)
Supplement: Additional file 2: Table S1. — The primers for MRPs. [file 12935_2015_190_MOESM2_ESM.docx]

Supplemental table 1 The primers for MRPs:

| Genes | sense | anti-sense |
| --- | --- | --- |
| MRP-1 | 5' CCGTGTTGGTCTCTGTGTTC 3' | 5' AAGTCGGCGGCGTAATTC 3' |
| MRP-2 | 5' GAGCACCAGCAGCGATTTC 3' | 5' TCCTCACCAGCCAGTTCAG 3' |
| MRP-3 | 5' TCTGGTCCCTAAAGGAAGAG 3' | 5' TTGAAGCAGGCACTGATG 3' |
| MRP-4 | 5' AGGCACTTCGTCTTAGTAAC 3' | 5' AAGGGCAGGAGAATGATTAG 3' |
| MRP-5 | 5' AGACTGTGGCAAGAAGAG 3' | 5' TCCAAAGGAAGGCTGAAC 3' |
| MRP-8 | 5' TGAGAGGAGCAATACAGAC 3' | 5' GATGGCAACCAGATTACAG 3' |

Supplemental Figure 1 legends

A. The mRNA of RhoGDI2 (left) and P-gp (right) in MKN-45/RhoGDI2 and MKN-45/GFP was detected by RT-PCR. Data was expressed as relative to GAPDH (mean ±SD) from three independent experiments; * *p*<0.05 vs MNK-45/GFP. B. Western blotting analysis of MRP-1 expression in MKN-45/RhoGDI2 and MKN-45/GFP.
